# "It doesn't do any harm, but patients feel better": a qualitative exploratory study on gastroenterologists' perspectives on the role of antidepressants in inflammatory bowel disease

**DOI:** 10.1186/1471-230X-7-38

**Published:** 2007-09-24

**Authors:** Antonina A Mikocka-Walus, Deborah A Turnbull, Nicole T Moulding, Ian G Wilson, Jane M Andrews, Gerald J Holtmann

**Affiliations:** 1 School of Psychology, University of Adelaide, Adelaide, SA, Australia; 2 School of Population Health & Clinical Practice, Discipline of General Practice, University of Adelaide, Adelaide, SA, Australia; 3 Department of Gastroenterology, Hepatology and General Medicine, Royal Adelaide Hospital, Adelaide, SA, Australia; 4 School of Medicine, University of Western Sydney, Sydney, NSW, Australia

## Abstract

**Background:**

Interest in psychological factors in patients with inflammatory bowel disease (IBD) has increased in recent years. It has even been proposed that treating psychological co-morbidities with antidepressants may control disease activity and improve quality of life. Despite this, there is no data on gastroenterologists' attitudes to, and experiences with, antidepressant therapy in patients with IBD.

**Methods:**

We conducted semi-structured interviews with 18 gastroenterologists associated with metropolitan teaching hospitals. Qualitative content analysis was used to examine their responses.

**Results:**

Seventy-eight percent of gastroenterologists had treated IBD patients with antidepressants for pain, depression and/or anxiety, and insomnia. Antidepressants were reported to be useful in improving psychosocial well-being, quality of life, and self-management of the disease by patients. However, in this group of gastroenterologists, there appears to be skepticism towards psychological disorders themselves or antidepressant therapy having a central role in either the causation of IBD or its clinical course. Nevertheless, these gastroenterologists were receptive to the idea of conducting a trial of the role of antidepressants in IBD.

**Conclusion:**

While the majority of specialists have treated IBD patients with antidepressants, there is considerable skepticism with regard to efficacy of antidepressive therapy or the role of psychological factors in the outcome of IBD patients.

## Background

Inflammatory bowel disease (IBD) is a term used to describe chronic, relapsing and remitting disorders of the gastrointestinal tract that are characterized by inflammatory changes of the gut wall and thought to be caused by an inappropriate immune response. The two common types of IBD are Crohn's disease (CD) and ulcerative colitis (UC). The prevalence of IBD has been estimated at 43,000 in Australia [1].

Like the majority of chronic diseases, IBD carries a physical, economic and social burden for sufferers but, perhaps most importantly; it also has serious psychological implications. Anxiety, depression and poor quality of life are all predictors of worse medical outcomes in many chronic conditions [2-5]. Moreover, psychological status is known to independently impact quality of life [6]. In general, the rate of common psychological disorders, such as depression and anxiety, is higher in chronically ill people than in the wider population and has been estimated at about 30% [7,8]. It is conceivable that in at least some of the chronically ill people the dual diagnoses (a somatic and a psychological problem) are etiologically distinct and each requires treatment on its own merits. Accurate diagnosis of depression becomes important because of the likely need for either effective drug therapy or a combination of drug therapy with psychotherapy. Treatment of depression is particularly important in light of its association with both medical noncompliance [9] and suicide [10,11].

It is not well understood whether it is as a consequence of their chronic disease or perhaps also from side effects of medication, IBD patients often suffer from psychological disorders that may, in turn, affect their response to standard treatment for IBD [2]. Patients who are diagnosed with both IBD and psychological disorders have been noted to have lower quality of life and more frequent relapses [2,12]. In addition to psychological disorders, patients with IBD are frequently also affected by irritable bowel syndrome (IBS) [13]. IBS is a chronic condition in which altered bowel habits and abdominal pain or discomfort are the predominant symptoms in the absence of any mechanical, infectious, biochemical or inflammatory changes in the gastrointestinal tract [14] using standard diagnostic tests. From 42% – 62% of CD patients and 33% of UC patients in remission suffer from IBS [13,15,16]. Quality of life and psychosocial well-being in these patients are therefore impaired regardless of whether their IBD is active or quiescent [17]. Moreover, as many as 93% of patients with IBS have a lifetime history of some psychiatric disorder [18].

For approximately 30 years, researchers have examined the effectiveness of various treatments for psychological disorders in IBD patients, although unfortunately much of this research is reported outside mainstream medical journals. In summary, this literature reveals that psychotherapy is in general ineffective in treating psychological disturbances in IBD patients, despite several different modalities having been applied [19]. However, pharmacotherapy with antidepressants, either as an alternative or adjunct to psychotherapy, has not yet been adequately explored (12). Nonetheless, some early reports of its efficacy in reducing both psychological and somatic symptoms, although unproven at present, seem promising [20]. Further research into the influence of antidepressants on the course of both CD and UC is therefore important, especially that antidepressants have been found to have a positive effect on symptoms and well-being of people with IBS [21]. However, before the larger sample studies take place, qualitative investigations exploring different aspects of this problem are highly recommended.

As one of the major barriers to implementing a change in disease management is the attitudes and habits of relevant clinicians, we sought to conduct a qualitative exploratory study into clinicians' views and practices on the use of antidepressants in IBD. The semi-structured interviews were designed to: 1) identify gastroenterologists' attitudes to the use of antidepressants in IBD; 2) investigate gastroenterologists' observations of the effects of antidepressants on IBD; and 3) explore gastroenterologists' attitudes towards further research in this area, especially towards possible randomized controlled trials.

## Methods

Data for this exploratory study were collected in October and November 2005 in two major South Australian metropolitan teaching hospitals, the Royal Adelaide Hospital (RAH) and the Queen Elizabeth Hospital (QEH). The study used purposive sampling where participants were selected based on their potential to provide data relevant to the research questions [22]. All participants were board recognized gastroenterologists with more than 2 years of professional experience as a specialist. None had any formal training in psychiatry. Interviews were conducted until the interviewer was satisfied that data saturation occurred. The researcher advertised the study during research meetings at the Department of Gastroenterology, Hepatology and General Medicine, at the RAH in September 2005 and at the Department of Gastroenterology and Hepatology, at the QEH in October 2005 and invited clinicians to indicate their willingness to participate. At the beginning of each interview each participant signed a written consent.

Digitally-recorded, standardized semi-structured interviews with 12 open-ended questions were undertaken with each gastroenterologist by the first author (AM-W). Demographic data were collected at the end of each interview. The interviews lasted up to half an hour with a median of 10 minutes. The recorded interviews were transcribed verbatim by the first author (AM-W). The draft of each interview was subsequently read and checked against the recordings by a research assistant. No major areas of disparity arose. Responses to each question were then collected and summarized by AM-W. Where it was appropriate to do so, responses were enumerated. Finally, AM-W confirmed the general organisation of data and their interpretation with NM.

Data were analyzed by qualitative content analysis [22]. The words and sentences relevant to the given question were identified by AM-W. Responses to questions were assigned to mutually exclusive categories where possible and subsequently coded by AM-W. Participants' responses were then summarized.

Ethical approval was obtained from the Royal Adelaide Hospital Human Research Ethics Committee. Each participant provided written informed consent.

## Results

Eighteen participants were recruited. Nine gastroenterologists participated at each site. Two Gastroenterologists associated with these departments did not participate due to their direct involvement in the study (JMA & GJH). This sample thus represents 78% (18 out of 23) of gastroenterologists working in these two hospitals, 66% (18 out of 27) of all the gastroenterologists working in the Central-Northern Adelaide Health Services, and 36.7% (18 out of 49) of all the gastroenterologists working in South Australia. Seventeen of the 18 participants were male, which is consistent with the gender mix for gastroenterologists in SA. Ten were born outside Australia. Their ages ranged from 33 to 64, with a median of 44. Their experience in gastroenterology ranged from two to 30 years, with a median of 16 years. Fifteen doctors completed their undergraduate studies in Australia. Ten doctors completed their postgraduate studies in Australia, five conducted them partly in Australia and partly abroad, and three completed postgraduate studies abroad. Participants reported treating between one and 12 IBD patients per week (with a mean of five patients).

### The role of antidepressants in IBD

All participants agreed that antidepressants were a useful medication for depression, IBS and various pain syndromes. The gastroenterologists often used antidepressants in chronically ill people due to the high prevalence of depression in this group. Two gastroenterologists believed antidepressants were overused, yet another two believed them to be underused. All participants were in agreement that antidepressants cannot be used as effective primary therapy in IBD and that no evidence exists in published research that antidepressants have an influence on the level of inflammatory markers. Most gastroenterologists, however, reported having successfully used antidepressants as an adjunct therapy. Interestingly, some gastroenterologists stated that psychological problems in IBD are easy to overlook and that in a number of patients, psychological issues are a significant concern.

### Reasons for using/not using of antidepressants in patients with IBD

Fourteen of eighteen participants had prescribed antidepressants or suggested using antidepressants in IBD patients. Reasons given for treating IBD patients with antidepressants were: pain, evidence of depression or significant mood disorder, which might include anxiety, depression, sleep problems, and gut symptoms despite being in quiscent phase (gut irritablity, IBS in IBD). Seven out of 14 gastroenterologists who had prescribed antidepressants had specifically treated depression or anxiety with antidepressants. Most (12 out of 14) of the participants who prescribed antidepressants to IBD patients, however, prefered referring depressed patients to their GP or to psychiatrist rather than being the initiators of this treatment. On the other hand, gastroenterologists felt comfortable with using antidepressants for pain, disturbed sleep patterns, and IBS symptoms in IBD. Those gastroenterologists who did not use antidepressants in IBD patients reported that this was either because they had not treated a depressed patient; did not believe depression was a significant problem in these patients; or did not see their role as detecting depression.

### The kind of antidepressants used and the result of the treatment

Participants had used classic tricyclics (e.g. amitriptyline, dothiepin, prothiaden, doxepin, imipramine, nortriptyline), selective serotonin reuptake inhibitors (SSRI) (citalopram, sertraline), and serotonin and noradrenaline reuptake inhibitors (SNRI) (mirtazapine) antidepressants in patients with IBD. However, with tricyclics they reported mainly focusing on treating somatic aspects of the disease e.g. pain, gut irritability, urgency of defecation. Tricyclics are, according to most of the participants, better researched in this area and have been found to be effective for IBS-like symptoms in IBD patients. Gastroenterologists often reported using the same antidepressants that they prescribe in patients with IBS. Only a few had tried treating depression and/or anxiety in their IBD patients with the newer antidepressive agents. Those who had used the newer agents felt that psychological problems responded well to this treatment.

### The treatment with antidepressants and the course of IBD

Ten of the 14 gastroenterologists who had prescribed antidepressant therapy did not perceive that antidepressants had any influence on the course of IBD. However, one of them explained his opinion in a following way: "I don't think it influences the activity of the IBD. The IBD is occurring in an individual and the individual's ability to manage the disease can clearly be improved by managing the accompanying mood disorder". Despite their opposition to the idea that antidepressants directly affect the course of the disease, many argued that the treatment with antidepressants seemed to improve patients' quality of life. In general, antidepressants were thought to help IBD patients in manage their disease, cope with unpleasant symptoms, improve patients' sleep patterns, reduce pain and gut irritability and help them look after their nutrition and diet. Moreover, the benefits of the treatment with antidepressants might be more visible on disease activity indices rather than on inflammatory activity markers. Two gastroenterologists who thought treatment with antidepressants had influenced the course of IBD reported that antidepressants helped in controlling exacerbations of IBD and reduced symptoms of IBD. These two gastroenterologists used amitriptyline, nortriptyline, citalopram, and sertraline.

Most participants who had used antidepressants in IBD patients (12 out of 14) believed that there was no difference in the effect of antidepressants on the disease between CD and UC patients. However, three doctors noticed that they mainly had treated CD patients with antidepressants, and not UC. One participant hypothesized that this was because of the earlier onset of CD compared to UC. In general, CD patients seemed to be treated with antidepressants for their psychological problems and pain whereas UC patients for pain and sleep disturbances. Nevertheless, the tendency was not very strong, as most participants did not differentiate between CD and UC in terms of their co-occurrence with depression and/or anxiety.

### Psychological treatments in IBD patients

Nine of 18 participants reported suggesting psychological treatment for their IBD patients. Some gastroenterologists reported that they referred their patients to psychologists, counselors, psychiatrists or general practitioners for psychotherapy. One doctor revealed he conducted some form of psychotherapy himself with his patients. Gastroenterologists also suggested hypnotherapy, relaxation and physical exercises to IBD patients. Gastroenterologists reported that they sought psychological treatment for their IBD patients when patients had symptoms of depression and/or anxiety; had chronic pain syndrome; psychosexual problems and problems with body image; or were opioid-dependent. The results of these treatments were, however, by and large unknown, as the gastroenterologists did not specifically follow up the patients with respect to psychological co-morbidities.

### Gastroenterologists' opinion on the feasibility of a trial with antidepressants in IBD patients

All 18 gastroenterologists said they would approve of their IBD patients' decision to enter a trial of antidepressant therapy as long as it was a properly conducted double blind randomized controlled trial.

## Discussion

This qualitative exploratory study was designed to investigate and describe the attitudes and practices of specialist gastroenterologists with respect to antidepressant and other psychological therapies in patients with IBD. Based upon this study, gastroenterologists are well aware of a high burden of anxiety and depression in IBD patients, although they do not routinely evaluate their patients for psychological co-morbidities. Many are open to the premise that treating anxiety and depression may improve patients' quality of life. The participants were, however, quite skeptical that  psychological disorders and/or antidepressant therapy had a central role in the  clinical course of IBD.

Although it is controversial, some investigators have proposed that the psyche may play a pivotal role in the causation of IBD [23-25], whilst others have proposed that psychological co-morbidities, especially anxiety and depression, may influence the clinical course of IBD [2]. As yet, the literature is unable to answer the question as to whether treatment of IBD with antidepressant therapy is of benefit [20], mostly due to the quality of available data: non-randomized, non-blinded, and non-controlled studies. However, antidepressants have been found to have anti-inflammatory properties by influencing production of anti-inflammatory cytokines [26,27]. There is also evidence that stress may trigger exacerbation of IBD by inducing pro-inflammatory responses [28] and that depression and anxiety may lead to more frequent relapses of IBD [2]. However, no trial devoted solely to the use of antidepressants in IBD has ever been conducted. Thus, one of the main aims of this investigation was to assess gastroenterologists' likely acceptance of a properly conducted clinical trial in this area. Indeed, the content analysis revealed that although the interviewed gastroenterologists used antidepressants in IBD to minimize symptoms and to treat insomnia, anxiety and depression, they would require strong evidence of specific benefit prior to accepting them as having a role in modifying the course of IBD.

In previous works on anxiety and depression in IBD, certain antidepressants have been suggested to have a specific benefit on the clinical course of IBD [29-33]. Unfortunately, none of gastroenterologists participating in the current study had used these antidepressants, which included paroxetine [29,33], bupropion [30,32] and phenelzine [31]. As the interviewed gastroenterologists were unable to confirm, or refute, the previously reported observations, the choice of an antidepressant for the evaluation in future trials is still unclear. Taking into account the opinions of the surveyed gastroenterologists, future research into the role of antidepressants in IBD should focus on both physical and psychological impact of antidepressants on IBD patients. If antidepressant therapy were shown to improve the clinical course of IBD further questions would need to be addressed. If all antidepressants were equally effective, one may infer that the psyche plays a major role in the course of IBD. On the other hand, if a specific antidepressant (or class of antidepressants) were more efficacious, our understanding of the inflammatory cascade in IBD itself may change. Future studies may, therefore, involve fairly complex designs with sub-group stratification and multiple arms as well as they would need to specify whether antidepressants are prescribed for IBD itself, for mood only or for pain related problems. Moreover, such studies could benefit from inclusion of a psychiatrist into a research team and research comparing the way gastroenterologists and psychiatrists prescribe antidepressants in patients with IBD could also be of interest.

Interestingly, during this study we also observed that some gastroenterologists had treated IBD patients with antidepressants in the same way they had treated IBS patients. This is not surprising given the symptomatic similarities of these two conditions [15] and the increasingly well documented overlap between these two diseases [13]. Twenty years ago IBS was thought to be a predominantly psychological condition with no inflammatory element, whereas we now have some evidence of pathological abnormalities in the gut [34,35]. By contrast, IBD is currently thought to be purely inflammatory, however, clinical observations in individual patients render it difficult to completely exclude psychological factors [36], and even the "objective" measures of severity such as the Crohn's Disease Activity Index (CDAI) are well known to be affected by the psyche by influencing ratings for "abdominal pain" and "general wellbeing". Moreover, as IBD and IBS commonly coexist, it is difficult to make categorical distinctions between psychological versus inflammatory mechanisms. It is, therefore, very important to explore various treatment options in the hope that informed discussion will prompt further research and improve standard medical care of these patients and thus, their quality of life. The fact that these surveyed gastroenterologists generally endorsed the concept of conducting a properly designed trial on the role of antidepressants in IBD and would encourage their patients to participate in such research is an important result in this regard.

## Competing interests

The study was funded by the Discipline of General Practice and the School of Psychology at the University of Adelaide, and the Department of Gastroenterology, Hepatology and General Medicine at the Royal Adelaide Hospital from International Postgraduate Research Scholarship. No industry sponsorship was involved. Authors are unaware of any conflicts of interest. However, Professor Deborah Turnbull would like to acknowledge that she has received research support from AstraZeneca Pty Ltd, Aventis Pharma Pty Ltd, Bayer Australia Ltd, and Pfizer Pty Ltd. Dr Jane M. Andrews would like to acknowledge that she has been a consultant for Schering Plough and Pharmatel Fresenius Kabi. Professor Gerald Holtmann would like to acknowledge that he has been a consultant for Abbott, Altana, AstraZenecka, Takeda, Janssen-Cilag, Steigerwald, Knoll, and Novartis. He has also received research support from Altana, Ardey Pharma, Deutsche Forschungsgemeinschaft, Zeria, and Novartis.

## Authors' contributions

AMW contributed to the conception of this manuscript, designed it, collected data, performed analysis and interpreted data, and wrote the first and final drafts. DT contributed to the conception of this manuscript, revised it critically and gave the final approval of the version to be published. NM contributed to the conception of this manuscript, revised the manuscript critically and contributed to the final draft. IW revised the manuscript critically and contributed to the final draft. JA assisted with study design, recruitment, revised the manuscript critically and contributed to the final draft. GH revised the manuscript critically and contributed to the final draft.

All authors read and approved the final manuscript.

## Pre-publication history

The pre-publication history for this paper can be accessed here:


